# Serogrouping and seroepidemiology of North European hantaviruses using a novel broadly targeted synthetic nucleoprotein antigen array

**DOI:** 10.1080/20008686.2017.1350086

**Published:** 2017-07-26

**Authors:** Bengt Rönnberg, Olli Vapalahti, Marco Goeijenbier, Chantal Reusken, Åke Gustafsson, Jonas Blomberg, Åke Lundkvist

**Affiliations:** ^a^ Section of Clinical Microbiology, Department of Medical Sciences, Uppsala University, Uppsala, Sweden; ^b^ Zoonosis Science Center, Department of Medical Biochemistry and Microbiology, Uppsala University, Uppsala, Sweden; ^c^ Laboratory of Clinical Microbiology, Uppsala University Hospital, Uppsala, Sweden; ^d^ Department of Veterinary Biosciences and Virology, University of Helsinki and Helsinki University Hospital, Helsinki, Finland; ^e^ Department of Viroscience, Erasmus MC, Rotterdam, The Netherlands

**Keywords:** Hantavirus, laboratory surveillance, suspension multiplex immunoassay, pathogen surveillance, megapeptide, synthetic antigen, viral haemorrhagic fever, zoonoses, emerging or re-emerging diseases

## Abstract

**Introduction**: Hantaviruses are globally distributed zoonotic pathogens. Great diversity and high antigenic cross-reactivity makes diagnosis by traditional methods cumbersome.

**Materials and methods**: ‘Megapeptides’, 119–120-mers from the amino terminus of the nucleoprotein of 16 hantaviruses, representing the four major branches of the hantavirus phylogenetic tree, were utilized in a novel IgG-based hantavirus suspension multiplex immunoassay (HSMIA) for detection of past hantavirus infections in 155 North European human samples. We compared HSMIA with established EIAs and focus reduction neutralization test (FRNT).

**Results and discussion**: The Puumala hantavirus (PUUV) component in the HSMIA gave concordant results with a PUUV IgG EIA in 142 sera from Northern Sweden (of which 31 were EIA positive, 7 borderline and 104 EIA negative, sensitivity 30/31 = 97%, specificity 104/ 104 = 100%, 134/135 = 99% concordance), with another immunoassay in 40 PUUV IgG positive sera from Finland (36/40 = 90% sensitivity), and was concordant in 8 of 11 cases with PUUV and DOBV neutralization titers, respectively. Two major IgG reactivity patterns were found: (i) a PUUV-specific pattern covering phylogroup IV and its serogroups B and C; and (ii) a Dobrava virus (DOBV)-specific pattern, covering the serogroup A portion of phylogroup III. In addition, we found several minor patterns with reactivity to only one or two megapeptides indicating additional hantaviruses infecting humans in the Swedish and Finnish populations.

**Conclusion**: The broadly reactive and rational HSMIA yielded results highly correlated with the established PUUV EIAs and the NT results. It is a sensitive and specific assay, which will be suited for efficient serosurveillance of hantaviruses in humans. Its use in animals should be further investigated.

## Introduction

Hantaviruses are globally distributed and can cause serious human infections – hemorrhagic fever with renal syndrome (HFRS) or hantavirus cardiopulmonary syndrome (HCPS) [1–5]. At present, 24 unique hantaviruses have been described and around 15 additional viruses await taxonomic acceptance [1]. New hantaviruses are currently discovered at a high rate in different mammals. Only a minority of the hantaviruses have been isolated from humans [2]. Hantaviral geographic distribution and host range are rather strict [3–14]. Host mobility, including human-mediated transportation, may be the reason why Seoul virus (SEOV), originally found in South East Asia, now has been found in rats and humans in Belgium, UK, France, Sweden, and the Netherlands [11]. These emerging zoonotic infections [15–18] need to be monitored. Current diagnostic methods [19] are mainly directed to a few viruses at a time, and thereby do not cover the great diversity of the hantaviruses.

We previously used synthetic, up to 30-mer, peptides in hantavirus serology [20,21] and multiplex serology with other microbes [22,23], later extended to 50- to 220-mers [24]. Long synthetic peptides (‘megapeptides’) have occasional defects in their sequences due to incomplete amino acid coupling, but most molecules will have a conformation that is similar enough to that of the complete native molecule, thus mimicking conformational epitopes. Posttranslational modifications are not included, but simplicity of creation and absence of unwanted cellular antigens partially outweigh this disadvantage. We here used megapeptides longer than 100 amino acids for hantavirus serology, using our Suspension Multiplex Immuno Assay (SMIA) technique [22–24]. It has earlier been shown that the first 107 amino acids of the amino terminus of hantavirus N proteins contain broadly cross-reactive immunodominant epitopes [20,25–27], dependent on two antiparallel beta helices [25–27]. The SMIA array contained synthetic 119–120-amino acid long peptides from the amino terminus of the nucleoprotein from all known major branches of the hantavirus phylogenetic tree [2], from phylogroup I/serogroup D; Thottapalayam virus (TPMV), from phylogroup II/serogroup D; Nova virus (NVAV), Laibin virus (LBV), from phylogroup III/Serogroups A and E; Dobrava virus (DOBV), Hantaan virus (HTNV), Andes virus (ANDV), Seoul virus (SEOV), Thailand virus (THAIV), Asama virus (ASAV), Oxbow virus (OXBV), Seewis virus (SWSV) and from phylogroup IV/Serogroups B and C; Puumala virus (PUUV), Tula virus (TULV), Sin Nombre virus (SNV), Laguna Negra virus (LANV), and Rockport virus (RKPV). The intention was to cover as many as possible of the known hantaviruses. We tested this array with sets of known hantavirus positive sera, exploring its capability to detect and type by using multivariate analysis. Using known PUUV and DOBV antibody positive sera we found that this indeed was possible. They displayed two major patterns of reactivity, conforming to either DOBV or PUUV specificity. We also got indications of infections with yet-to-be discovered hantaviruses in blood donors and other persons from the Nordic countries.

## Materials and methods

### Synthetic peptides

**Figure 1.**
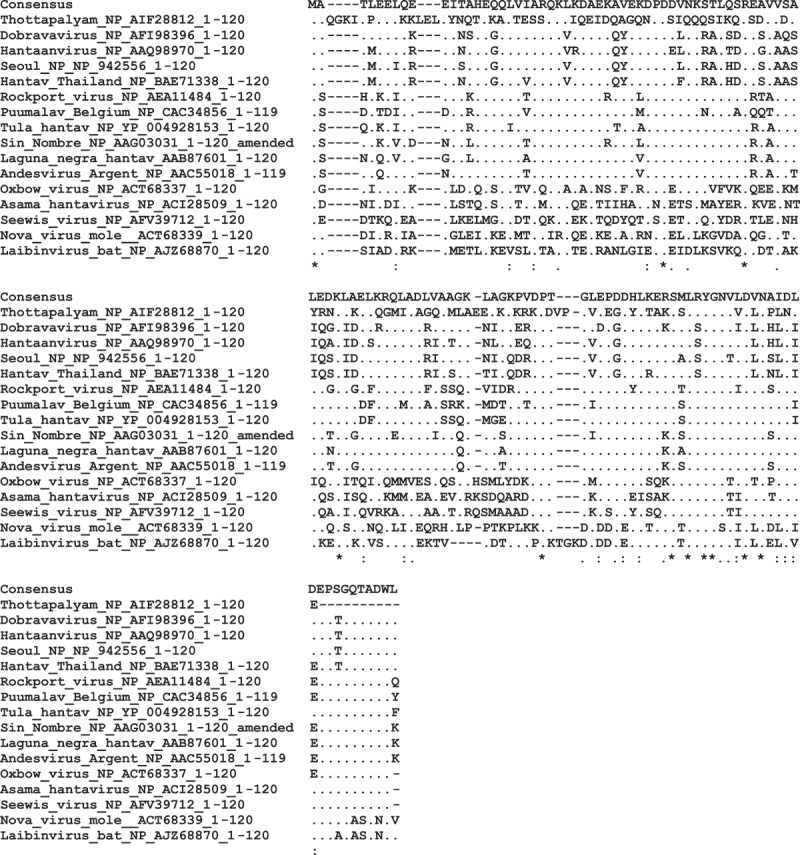
Megapeptides from the N terminus of hantavirus N proteins used in the present work. Peptide sequences were aligned by Muscle. A majority consensus is shown above the alignment. Positions with identity to the consensus are shown as dots. Conservation is also shown with symbols below the alignment, i.e. * = Fully conserved, : = semiconserved and . = conservation with exchange of similar amino acids. GenBank Ids are shown for each sequence. The tree (phylogram) of Figure 2 was derived from this figure.

The peptide sequences are shown in Figure 1. All had the spacer NH_2_-PEG_6_-His_6_-PEG_6_ at their amino terminus, where PEG_6_ is hexaethylene glycol and His_6_ is hexahistidine (enabling coupling control via anti-His antibodies). The peptides were synthesized under conditions optimized for maximum coupling yield of long peptides, see e.g. [24]. Lyophilized peptides were dissolved in dimethyl sulfoxide (DMSO) (Sigma D2650).

**Figure 2.**
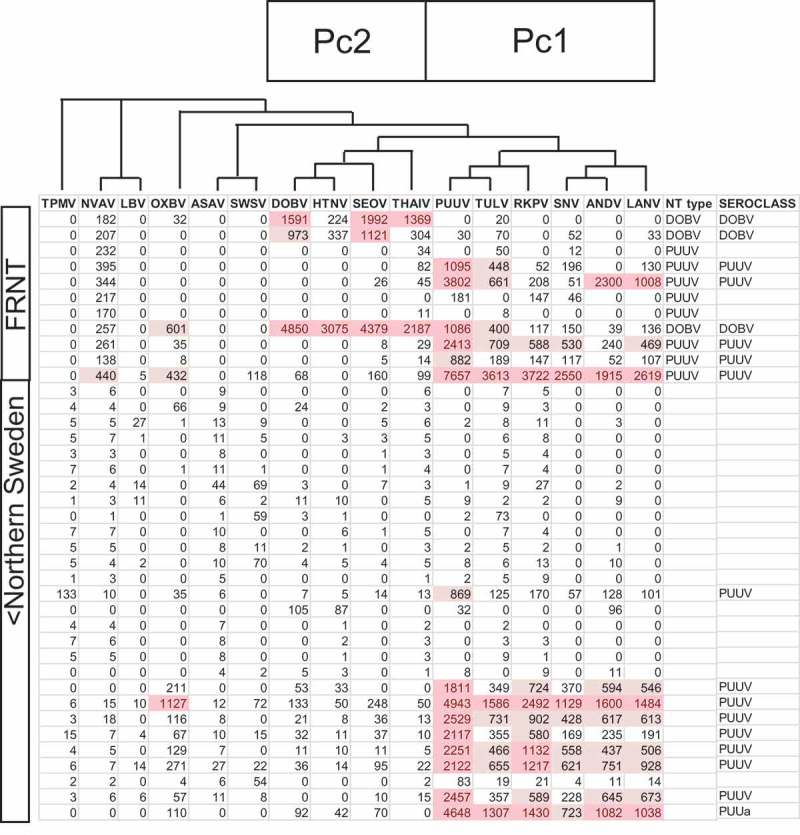
Part of the total result table. Each row represents one serum. The first 11 rows are from the FRNT collection, the subsequent 28 are a portion from the North Swedish collection (totally 104). Megapeptides (heading) and their results (MFI) are arranged in phylogenetic order, according to a dendrogram based on megapeptide protein similarity obtained after Muscle alignment at the European Bioinformatics Centre (EBC) home page. The megapeptide components which contributed most to principal components 1 and 2, ‘Pc1’ and ‘Pc2’, respectively are also indicated. Results were automatically classified (shown in column ‘seroclass’) as described in the text. All results are averages of two determinations and subtracted with the MFI of the ‘naked’ bead. MFI values higher than 400 MFI and 1000 MFI have been highlighted, in a graded fashion. Nova peptide reactions were relatively strong in the FRNT sera. We assume this was due to heat inactivation.

The epitope prediction tools at the Immune Epitope Database [28] and 3D structures found in the Protein structure DataBase (see e.g. PDB id 2K48) [29] were utilized for optimization of megapeptide design.

### Coupling of peptides to magnetic Luminex beads

The peptides were coupled to Luminex carboxylated magnetic beads (MagPlex-C microspheres, Luminex Corp, TX, USA) according to the manufacturer’s protocol ‘Sample protocol for two step carbodiimide coupling of protein to magnetic beads’ (http://cdn2.hubspot.net/hub/128032/file-213083097-pdf/Luminex-xMap_Cookbook.pdf).

The coupling was made with 200 µl of the stock microspheres, containing 1.25 × 10^7^ beads per ml, or a smaller proportion of this volume. In the latter case, all volumes in the recipe were decreased proportionally. After the coupling, beads were incubated with 0.5 ml of PBS containing 0.05% (v/v) Tween 20 and 50 mM Tris in the dark for 15 min on a rocking mixer at room temperature to block unreacted carboxyl groups with primary amines. The microspheres were then washed once with 0.5 ml StabilGuard (SurModics, Eden Prairie, MN, USA, #SG01-1000) using the magnetic separator. The bead pellet was finally resuspended in 400 µl StabilGuard. This created a bead mixture consisting of 6250 beads/µl. The coupled beads were stored at 4°C in the dark.

### Serum samples

#### Sera typed by focus reduction neutralization test (FRNT)

These samples (*n* = 11) were described in a previous study on hantavirus infections in the Netherlands [30]. These sera were initially identified by screening by IFT and EIAs and subsequently confirmed (serotyped) by FRNT. This study was exempted from ethical review of human subject research by the Medical Ethical Review Committee of the Erasmus MC Medical Centre, University of Rotterdam. All data have been anonymized and are not attributable to individual patients.

#### Sera from Northern Sweden

Sera from Northern Sweden (*n* = 142) were obtained as part of a serological survey for PUUV-reactive antibodies [31]. All samples were from healthy adults, and were obtained with ethical permission number Dnr2011/819-31/3). All samples were initially tested by a Puumala IgG EIA, see below.

#### Sera from Finland

Sera from Finland (*n* = 40) were sent to Helsinki University Central Hospital Laboratory Service (HUSLAB, Department of Virology and Immunology, Zoonosis Unit, Helsinki, Finland) for PUUV IgM and IgG analysis for suspicion of PUUV infection and had positive IgG but negative IgM result. The collection for the study was performed under HUSLAB’s research permission for OV (granted on 14 June 2013). The PUUV IgM and IgG reference results are accredited (SFS-EN ISO/IEC 17025 and SFS-EN ISO 15189; Finnish Standards Association). The IgM test (data not utilized in the present paper) was an IgM capture enzyme immunoassay based on recombinant PUUV-N produced in the baculovirus system [32]. The IgG test was an in-house immunofluorescence assay (IFA) based on PUUV-infected acetone-fixed Vero E6 cells [33,34].

#### Blood donor samples

Blood donor samples (*n* = 89) from Uppsala, obtained anonymously with recorded donor consent according to the Swedish Biobank law and ethical consent UPS_01_367, from the Uppsala University Hospital blood bank. The Swedish Biobank law states that sera obtained with donor consent may be reused for developmental purposes if the purpose is related to the original purpose of taking the sample.

### Serologies

#### Hantavirus suspension multiplex immunoassay (HSMIA)

Fifty µl of serum diluted 1/20 in PBS, pH 7.4, containing 0.05% (v/v) Tween 20, 50 mM Tris and 2% (v/v) Prionex (Sigma-Aldrich #81662) (PBSTP) was added to wells of a round bottom 96-well microtiter plate (Greiner #104650). Fifty µl of a vortexed and sonicated bead mixture consisting of 25 beads/µl was then added to each well. The plate was then incubated in the dark with gentle rotation for 1 h at 37°C. Wells were washed once with PBS using a magnetic plate separator (Life technologies #A14179). Beads were resuspended in 50 µl of PBSTP and 50 µl of biotinylated protein G (Pierce, article nr 29988) (4 µg/ml PBSTP) was added to each well. After 30 min of incubation in the dark with rotation at 37°C, the wells were washed once in PBS. Beads were resuspended again in 50 µl of PBSTP. Fifty µl of streptavidin-phycoerythrin (SA-PE) (InVitrogen-Thermo-Fisher, article nr S-866) (4 µg/ml in PBSTP) was added per well. The plate was incubated in the dark with gentle rotation for 15 min at 37°C. Beads were washed once in PBS before they were resuspended in 100 µl of PBS and analyzed in a Luminex-200 (Luminex corporation, Austin, Texas, USA) instrument following the manufacturer’s instructions. A minimum of 100 events per bead set were read and the median value obtained for each reaction event per bead set. A sample volume of 75 µl was analyzed for each sample. All samples were analyzed in duplicate and average readings calculated. To detect any antibody binding to the beads themselves, a naked non-peptide-containing (‘blank’) bead was included. A control His_6_ bead was also included in the SMIA reaction. The His_6_ tag allowed monitoring of coupling of the megapeptides to the magnetic beads using anti-hexahistidine antibodies (Antibodies on line, ABIN100493). We did not observe false positive reactions due to anti-His_6_ in the sera tested for this paper. One negative serum control, where PBSTP instead of serum was added, was also used in all experiments. A cutoff was first calculated as the PUUV average MFI of negative samples (OD < 0.1 in Puumala EIA) + 3 standard deviations, resulting in 108 MFI. In earlier SMIA work, we used a more rigorous cutoff of 200 MFI [22,23]. This higher value was subsequently used. The reproducibility of HSMIA was determined in 40 PUUV positive sera from Northern Sweden. They gave a standard deviation of 11% after retesting.

#### Comparative serological tests

A PUUV IgG EIA, based on Mab-captured baculovirus-expressed PUUV nucleocapsid protein (N), was performed as previously described [35]. The Finnish sera were tested with a µ capture assay [35] for PUUV IgM, and were positive for PUUV IgG according to an immunofluorescence (IFA) against PUUV and a TR-FRET assay [36].

#### Focus reduction neutralization test (FRNT)

To confirm hantavirus-reactivity and -specificity, a focus reduction neutralization test (FRNT), a highly virus species-specific technique for hantavirus serology, was performed as previously described [35,37,38]. Briefly, serially diluted serum samples were mixed with virus and incubated on confluent Vero E6 cell monolayers in six-well tissue culture plates for 7–12 days depending on the hantavirus. Monkey or rodent anti-hantavirus polyclonal sera were used as primary antibodies (35b, 35c) and peroxidase-labelled goat anti-human or anti-rabbit IgG (BioRad Laboratories, Hercules, CA, USA; and Jackson Laboratories, Bar Harbor, ME, USA) as conjugates, followed by 3, 3ʹ, 5, 5ʹ-tetramethylbenzidine substrate (Sigma, Stockholm, Sweden). An 80% reduction of the number of foci was used as cut-off for virus neutralization.

#### Statistical evaluation, computerized handling of results and bioinformatics

Principal Component Analysis (PCA) was performed using the multivariate program Unscrambler (CAMO AS, Bergen, Norway). Values from 282 samples, each comprising 16 observations, were subjected to the exact singular value decomposition PCA method. No missing data occurred in the dataset. All results were subtracted, averaged and collected in a .dbf table with columns in phylogenetic order, and categorized, using a program (‘SeroClass’) written by JB in Visual Foxpro. Categorization followed the two major principal components and the three minor patterns not detected by PCA. The PCA defined PUUV and DOBV defined patterns included weights (PCA loadings), for each megapeptide pattern component. The sum of MFI times weight for each megapeptide had to reach a threshold of 300. The inclusion criterion for the minor patterns was that the first MFI of the pattern-defining megapeptide was stronger than MFIs of other megapeptides for that serum, and higher than 300.

## Results

### Design of megapeptides and creation of a broad hantavirus serological array

The N terminus of the N protein was chosen as the antigenic target because of its earlier proven high antigenicity, high conservation and high cross-reactivity. Before megapeptide synthesis, the surface availability of epitopes of crystallized hantaviral N proteins was evaluated using the Discotope program at IEDB [28,39]. The N terminus up to about amino acid 120 forms a prominent, highly exposed, protrusion (not shown), predicted to contain conformational and linear epitopes, corroborated by experimentation [32,40,41].

The absence of glycosylation in the N protein was also judged to be favorable for using synthetic antigen mimicks. We therefore synthesized NH_2_-PEG_6_-His_6_-PEG_6_ 119–120mers from the N terminus of 16 hantaviral N proteins (Figure 1), intending to cover all known hantavirus phylogroups. At least one representative of the major phylo- and serogroups was included (Figure 1). The multiplexing capability and the high precision of quantification of the SMIA results allowed us to create a rational hantavirus serological grouping and detection system, HSMIA.

The HSMIA system allowed us to process the 282 samples in a short time, generating 16*282 = 4512 IgG reactivity values. Initial titrations indicated that a serum dilution of 1/20 gave strong reactions of up to about 5000 MFI and background values of around 40 MFI, which after background subtraction (subtraction of the value of the naked bead) became close to 0, yielding both high sensitivity and specificity.

### Analysis of groups of sera

We first analyzed 11 sera that had earlier been typed by neutralization. Three were DOBV-specific and eight PUUV-specific, giving NT titers of >40 up to 2560. The HSMIA results followed the neutralization results, with some exceptions (Figure 2). The three sera, which had a high DOBV NT titer also showed a DOBV HSMIA pattern, while five out of eight sera that were PUUV FRNT-specific also showed a PUUV HSMIA pattern. The remaining three PUUV FRNT-specific sera only reacted weakly (below 200 MFI) with PUUV, TULV, and THAIV in HSMIA. The FRNT sera had been heat inactivated, and gave higher reactions with the naked beads, for unknown reasons also with the Nova megapeptide. Results by the universal positive control – a rabbit anti-hexahistidine serum (not shown), assured us that the hantaviral megapeptides were antigenically active.

We then analyzed 104 sera from Northern Sweden which had previously been found to be negative (OD < 0.1) using the PUUV virus IgG EIA [42] (Table 1). The bottom 28 rows in Figure 2 are from the serosurvey in Northern Sweden. One of these sera had an ‘Asama’ (ASAV) reactivity by HSMIA (727 MFI; see below). The remaining 103 became negative (with an MFI pattern sum cutoff set to 300 MFI). For example, the average MFI of the DOBV megapeptide was 6.3 (SD 15.8) after background subtraction and 41.0 (SD 45.3) before subtraction, and of the PUUV megapeptide 9.0 (SD 29.0) after subtraction, and 44.0 (SD 44.9) before subtraction. The concordance for the IgG reactivity of the two megapeptides with PUUV IgG EIA for negativity was 103/104, i.e. 99%.

**Table 1.** Overview of sera and results.

Serum collection	No. of sera	Selected sera	NT titer (focus reduction)	Puumala EIA	Puumala component of HSMIA	Dobrava component of HSMIA	HSMIA pattern
FR-NT panel	11	Selected sera (SIIDC)	Dobrava titer≥160	n.d.	0–1086 MFI	973–4849 MFI	DOBV (*n* = 3)
			Puumala titer≥160	n.d.	882–7657 MFI	0–68 MFI	PUUV (*n* = 3)
			Dobrava < 160, Puumala < 160	n.d.	1095–3803 MFI	0 MFI	PUUV (*n* = 2)
					0–181 MFI	0 MFI	None (*n* = 3)
Northern Sweden	104	Serosurvey, SIIDC	n.d.	OD < 0.1	0 MFI	11 MFI	ASAV (*n* = 1)
					0–181 MFI	0–105 MFI	None (*n* = 103)
	7		n.d.	OD ≥ 0.1 and OD<0.2	322–869 MFI	0–52 MFI	PUUV (*n* = 3)
					0–3 MFI	0 MFI	None (*n* = 4)
	31		n.d.	OD ≥ 0.2	812–5768 MFI	0–163 MFI	PUUV (*n* = 30)
					83 MFI	0 MFI	None (*n* = 1)
Finland	40	Samples received for diagnosis	n.d.	Positive	229–8478 MFI	0–249 MFI	PUUV (*n* = 36)
					0–91 MFI (incl. 2 bead binders)	0–6 MFI (incl. 2 bead binders)	None (*n* = 4)
Uppsala, Sweden	89	Blood donors	n.d.	n.d.	460–4908 MFI	4–43 MFI	PUUV (*n* = 3)
					4–69 MFI	0–8 MFI	ANDV (*n* = 2)
					5–16 MFI	0–9 MFI	SWSV (*n* = 2)
					0–192 MFI	0–215 MFI	None (*n* = 82)
Total no. of sera	282						

OD, Optical density in EIA; SIIDC, Swedish Institute for Infectious Disease Control; n.d., Not done; MFI, Median Fluorescent Intensity in HSMIA, after background subtraction

**Figure 3.**
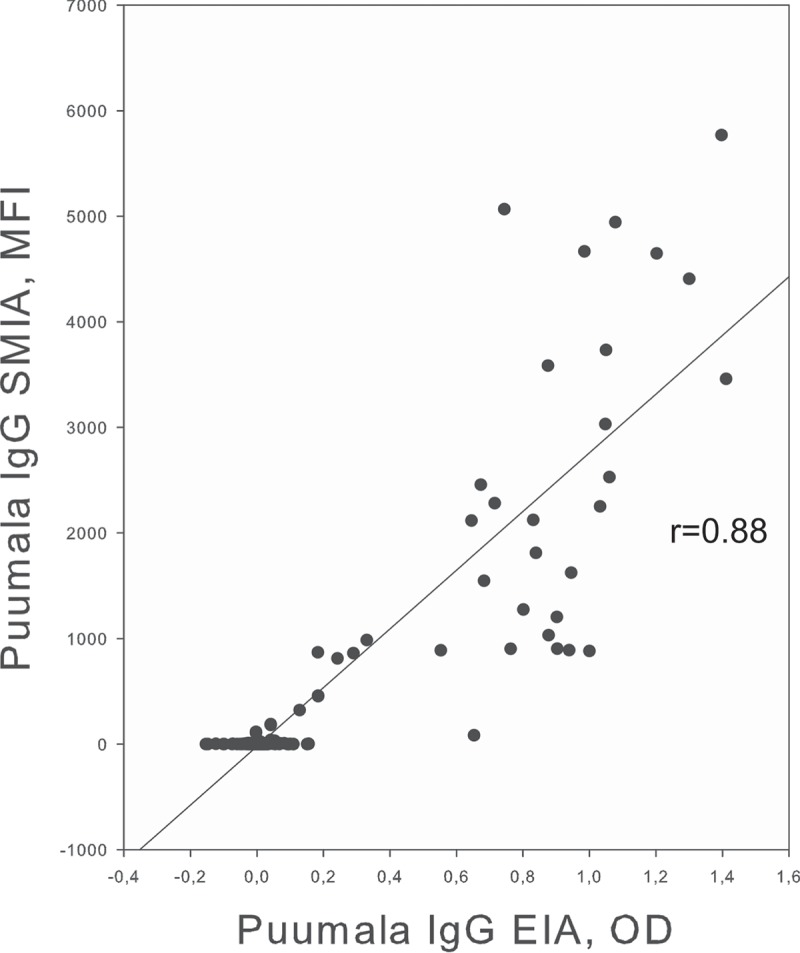
Correlation between HSMIA PUUV MFI and SIIDC PUUV EIA OD, for the 134 samples from Northern Sweden. X axis: SIIDC PUUV EIA OD, Y axis: HSMIA PUUV megapeptide MFI after background subtraction. A regression line (yielding a linear regression coefficient of 0.88) is also shown.

Subsequently we analyzed 31 samples from Northern Sweden by an EIA OD of over 0.2 (Table 1). A total of 30 reacted with the PUUV megapeptide with an MFI of over 200 (after background subtraction), giving a concordance of 30/31 = 97%). The correlation between the two methods is shown in Figure 3. All 30 were classified as PUUV-specific (see below). The 31st serum (EIA absorbance 0.653) reacted only weakly, below 200 MFI, with PUUV (83MFI) and SWSV (54 MFI). Calculating sensitivity and specificity from Table 1 gave a HSMIA PUUV component sensitivity versus PUUV IgG EIA EIA of 30/31 = 97%, and a specificity of 104/104 = 100%. MFI of the PUUV component correlated clearly (linear regression coefficient (r) = 0.88) with the OD of the PUUV IgG EIA (Figure 3).

When the Finnish ‘PUUV Immune’ (IgG positive) sera were analyzed by HSMIA, 36 of 40 became positive against the PUUV component. The 36 also had a ‘PUUV-specific’ pattern as defined below. Of the four remaining sera, two were strong ‘bead-binders’ (2428 and 681 MFI, respectively, with naked beads; see Materials) leading to a probable oversubtraction of the naked bead background value. Thus, 36 of the 38 (95%) samples which could be adequately analyzed were detected by HSMIA.

Three of 89 Uppsala blood donors were found PUUV component positive. All three had a ‘Puumala’ pattern (see below). However, two other sera were ‘Andes’ (ANDV) positive, and a further two sera were ‘Seewis’ (SWSV) positive. These reactivity patterns are defined below.

### Definition of major and minor hantaserological patterns

**Figure 4.**
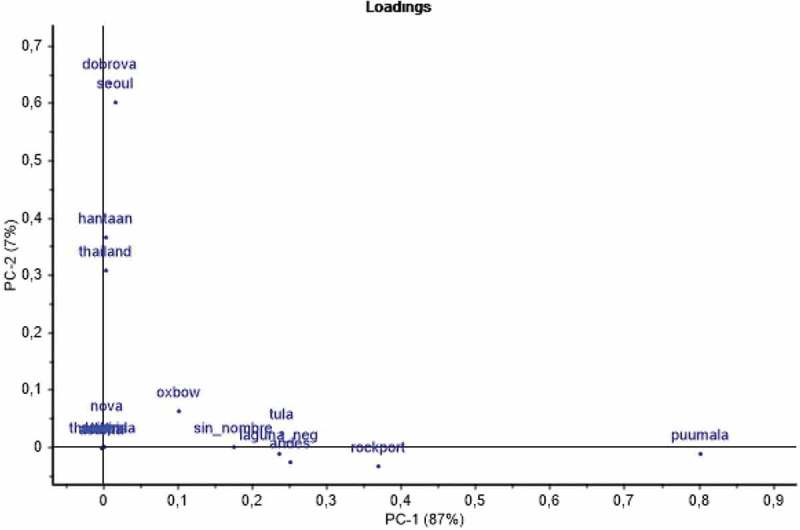
Principal component analysis. The two first principal components are PUUV (PC-1) and DOBV (PC-2) specific. ‘Loadings’ depict the influence of megapeptides for each principal component (Pc1 and Pc2).The two principal components together explained 95% of the variation in the dataset (not shown). The figure was produced by the multivariate program ‘Unscrambler’.

PCA was performed on all 282 results from the panels. The positive serological results fell into two major categories, principal component 1 (Pc1), and principal component 2 (Pc2) (Figure 4). The two principal components were orthogonal with respect to each other, i.e. they varied independently. Pc1 corresponded to PUUV-specific reactions, corroborated by the PUUV-specific neutralization of some of these sera and a high correlation with the PUUV EIA. Pc2 corresponded to DOBV-specific reactions, corroborated by the DOBV-specific neutralization of some of these sera. A total of 77 sera had a Pc1 (PUUV-specific) pattern of reactivity. Three sera had a Pc2 (DOBV-specific) pattern of reactivity (Table 1, Figure 2).

**Figure 5.**
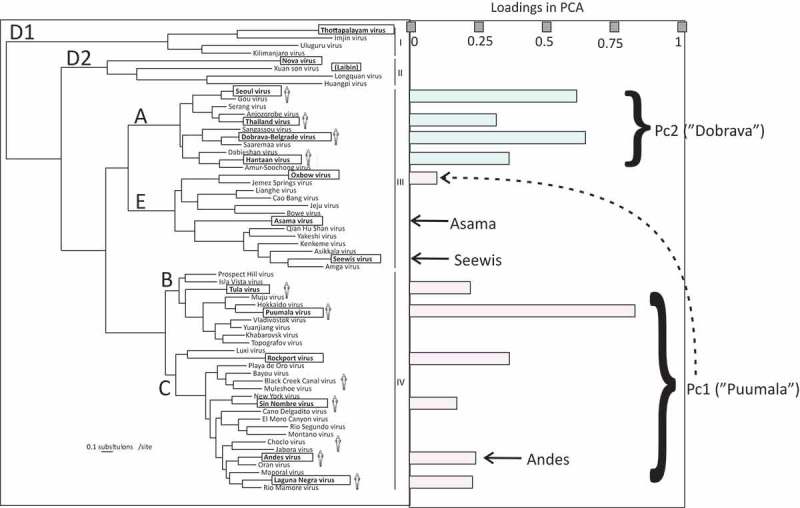
Distribution of hantavirus N protein megapeptides (bold face and boxed) in a hantavirus S segment phylogenetic tree. The tree was redrawn from that of Holmes and Zhang [2]. Viruses known to give disease in humans are shown with a human figurine. Phylogroups I–IV and serogroups A–D [26,27] are indicated. Laibin virus (Phylogroup II) was recently discovered [42]. The distinction between serogroups D1 and D2 was introduced in this paper. PCA ‘loadings’ indicate the degree of influence for each antigen in the respective principal component. They are here plotted according to phylogenetic relationship. Judging from the loadings of megapeptide antigens, Pc1 covers serogroups B and C while Pc2 covers serogroup A. However, Oxbow virus (Serogroup E) gave a minor contribution to Pc1 (stippled arrow). Minor more solitary patterns are shown with arrows. Andes reactivity was both observed within the PUUV pattern and as a minor more solitary pattern.

The distribution of Pc1 megapeptide serological reactions largely followed the distribution of a subset of phylogroup IV [2], serogroup B [26], while Pc2 megapeptide reactions were confined to phylogroup III [2], serogroup A [26] (Figures 4 and 5).

**Figure 6.**
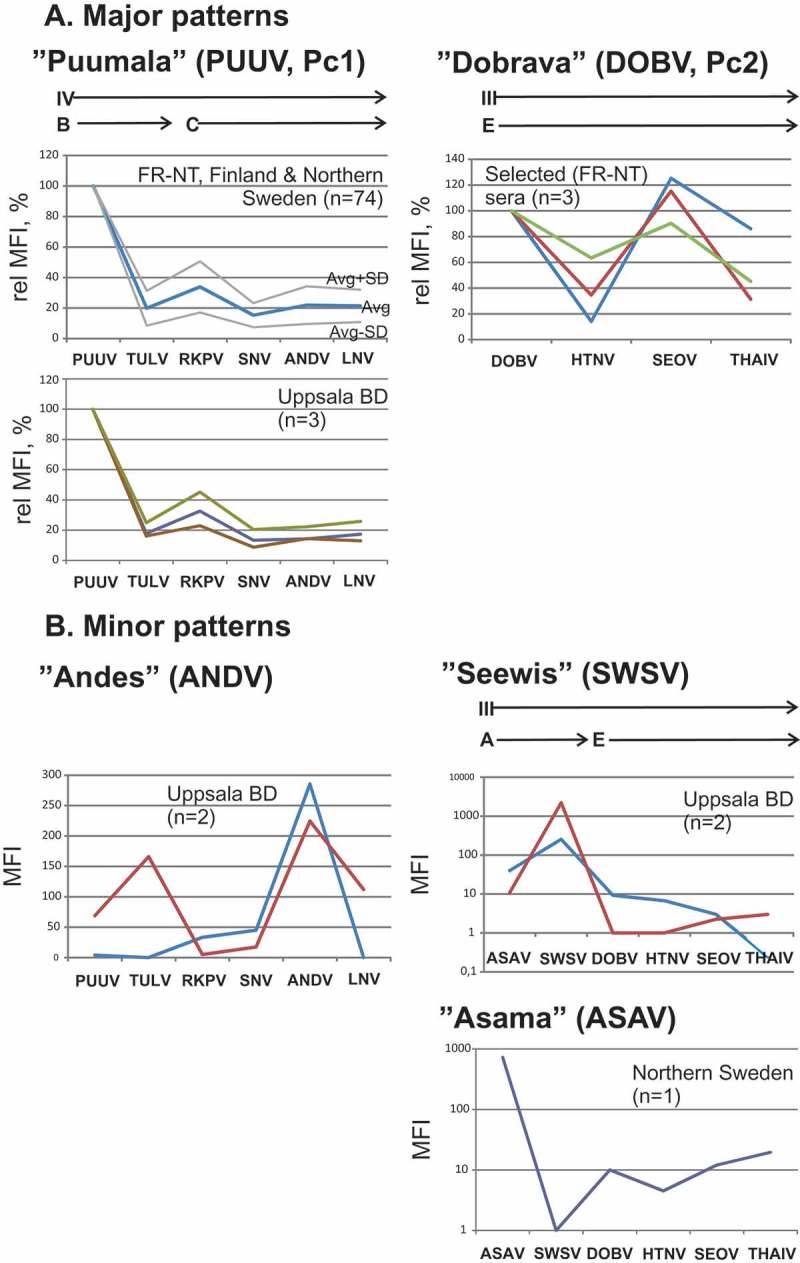
Detailed presentation of major and minor HSMIA patterns. Roman numerals refer to phylogroups, alphabetical characters to serogroups of Megapeptide sequences. X axis = contiguous hantavirus megapeptides (order as in Figures 1 and 4) belonging to a serological pattern. Y axis: either relative MFI, in % of the strongest reacting megapeptide or MFI, in linear or logarithmic representation. The PUUV pattern is shown with standard deviations (SD), where the red line shows the average relative MFI + 1 SD, and the green line shows the average − 1 SD.

Aside from the two major patterns there were minor patterns that occurred outside of them. The patterns can be studied in detail in Figure 6, where MFI of the two major patterns were normalized to PUUV and DOBV megapeptide reactivities, respectively (Figure 6(a)).

## Discussion

Serology has advantages as compared to nucleic acid (PCR)-based systems. Our SMIA system is simple to perform, can easily be automated, and uses only a few microliters of serum. Desirable extensions of the HSMIA work are testing of sera from all major continents, and adaptation for IgM determination. PCR is an important adjunct to HSMIA but is more laborious, requires extraction of a larger volume of sample, and may require sequencing for typing. Moreover, many hantavirus infections give a rather short period of viremia, which may not reach high copy numbers, see e.g. [43,44], thus serology is required for the diagnosis of acute hantavirus infections.

The disadvantages of HSMIA relative to EIA are: (i) It requires a Luminex flow meter, which is not present in all laboratories; and (ii) the color-coded beads are relatively expensive, a drawback in the early period of development when many antigens need to be tested. Otherwise, HSMIA offers multiplexity and serotyping, is more economical in terms of antigen (10–50 fold less is used per sample) and sample volume (only 1–5 uL per assay) use.

### Hantaviruses as a target for surveillance and diagnosis

Hantaviruses cause zoonotic infections and are a serious global medical problem. Appropriate diagnostic systems are not present in many parts of the world. The great number of newly discovered hantaviruses, which may or may not give human infections, requires appropriate surveillance and diagnostic systems. We took advantage of the high conservation and antigenicity of the N terminus of the N protein. This, coupled with the facile production of megapeptides, allows an efficient detection of past infections of diverse hantaviruses. We are now evaluating the generality of this serology in a broader geographic context than analyzed here.

### HSMIA and its design

We used a long spacer, which included His_6_, to ensure optimal presentation of N protein epitopes in the megapeptides. The N protein contains more or less group-specific epitopes distributed over its entire length [20]. Truncated N protein antigens were previously used [20,45,46]. It was previously suggested to be a suitable antigen for serotyping [26]. We strived for inclusion of the most cross-reactive epitopes [25–27,45] that would allow detection of antibodies to widely differing hantaviruses. At least one representative of all four known phylogroups [2] was therefore synthesized. Coupling efficiency was monitored throughout the megapeptide synthesis. The SMIA system is useful for large scale serosurveys in humans and animals because of the use of protein G, which can bind to a large variety of mammalian IgGs. Calculation of median fluorescence intensity (MFI) of 100 determinations gave a great precision, facilitating distinction of serological patterns. The background was low. Two of the 282 sera gave an MFI exceeding 200 (‘bead binders’). Such occasional non-specfic reactions [47,48] were reduced by background subtraction.

### Relevance for detection and seroepidemiological surveillance of emerging hantaviruses

The HSMIA patterns conformed to the serotyping of the same sera by FRNT with DOBV and PUUV. The patterns largely followed phylogenetic relatedness. Patterns occurred within or between related serogroups. The PCA results were clear-cut, with two principal components (DOBV and PUUV) that were orthogonal to each other, i.e. with minimal overlap. The two patterns conformed largely to the FRNT and EIA results. The ability of the system of 16 megapeptides to distinguish at least two major seroreactivity patterns, of DOBV and PUUV infections, respectively, makes it likely that infections with other hantaviruses can also be detected and broadly typed with this system. Once a pattern has been identified, it can easily be added in the automatic pattern recognition algorithm. A multiplex method like SMIA, where all antigens compete for antibody binding in the same solution, inherently has a high precision. This should allow the definition of antibody reactivity patterns for many different hantaviral species, also for hitherto unknown ones. The influence of host variation, including histocompatibility antigens, on the patterns is unknown, but the use of a broad vertebrate IgG detection (Protein G) allows this to be rationally tested.

We have currently no explanation for the weak atypical PUUV pattern in the nucleoprotein-based HSMIA in the three sera that had PUUV specificity in FRNT. FRNT depends on other epitopes than those present in the N terminus of the nucleoprotein. The chosen nucleoprotein portion is conserved and variation in it is an unlikely cause of the result. Reassortment between virus genetic segments [49], time- or HLA-dependent epitope recognition could be involved.

Minor patterns were also observed. Two, ANDV and SWSV, occurred in blood donors. They could represent spurious immunological cross-reactions occurring in a few humans, but may indicate that a few Uppsala blood donors have been infected with hitherto unknown or underdiagnosed hantaviruses, during international travel or inside Sweden. HSMIA is tailored for hantavirus surveillance, allowing attempts to correlate serological patterns to newly detected, rare or emerging viruses and to disease.

For example, SEOV was recently found in rats in the Netherlands and Sweden [12,50]. Finnish sera reactive with a SWSV antigen in a singleplex EIA, ascribed to PUUV cross-reactions were recently described [51]. Our SWSV reactive sera had a distinct pattern with little PUUV megapeptide reactivity, indicating a SWSV-like origin of this pattern. This could be further studied using serology with less cross-reactive antigens, like glycoproteins, of SWSV, ASAV and ANDV.

## Conclusions

The simplicity as well as comprehensive and easily extensible hantaviral coverage of HSMIA and its ability to measure antibodies in a wide range of mammals makes it an attractive system for global hantaviral surveillance. Megapeptide-based arrays should also be useful in other virus families with a mixture of linear and conformational non-glycosylation dependent epitopes. It is likely that PCA and HSMIA will aid in the detection of new hantaviral serological patterns and perhaps also in the detection of novel hantaviruses. It should be followed up with nucleic acid (PCR- or next generation-sequencing) and other serological tests, such as neutralization assays.
